# *Lactococcus lactis* as a New Strategy for Oral Vaccination: Current Insights and Future Perspectives

**DOI:** 10.3390/pharmaceutics18030307

**Published:** 2026-02-28

**Authors:** Jonnathan Grossolli-Galvez, Mónica Imarai, Jorge A. Soto, Abel E. Vasquez

**Affiliations:** 1 Laboratorio de Inmunología, Departamento de Biología, Centro de Biotecnología Acuícola, Facultad de Química y Biología, Universidad de Santiago de Chile, Santiago 91700022, Chile; jonnathan.grossolli@usach.cl (J.G.-G.); monica.imarai@usach.cl (M.I.); 2 Translational Immunology Laboratory, Department of Biological Sciences, Faculty of Life Sciences, Universidad Andres Bello, Santiago 8370146, Chile; jorge.soto.r@unab.cl; 3 Center for Research on Pandemic Resilience, Faculty of Life Sciences and Institute of Public Health, Universidad Andres Bello, Santiago 8370146, Chile; 4 School of Veterinary Medicine, Faculty of Medicine and Health Sciences, Universidad Mayor, Santiago 8580745, Chile; 5 Center for Biomedicine, Universidad Mayor, Santiago 8580745, Chile

**Keywords:** *Lactococcus lactis*, GMO regulatory frameworks, mucosal vaccines

## Abstract

*Lactococcus lactis*, a safe food-grade lactic acid bacterium, has attracted increasing attention as a live biotherapeutic platform for mucosal vaccine development. Its genetic simplicity, absence of endotoxins, and availability of well-characterized inducible systems have enabled controlled expression and delivery of heterologous antigens and therapeutic molecules. This review highlights recent advances in the use of genetically modified *L. lactis* for mucosal immunization, focusing on expression technologies, routes of administration, and immune mechanisms relevant to protection or tolerance. Preclinical studies demonstrate its capacity to induce both mucosal and systemic immune responses against diverse pathogens, underscoring its potential as a safe and versatile vaccine chassis. Remaining challenges include regulatory harmonization, biosafety concerns, and the need for standardized manufacturing and evaluation frameworks. Together, these developments position *L. lactis* as a promising candidate for next-generation mucosal vaccines and live biotherapeutic products.

## 1. Introduction

The term “probiotic” was first introduced in the 1960s and later formalized by the World Health Organization (WHO) and Food and Agriculture Organization (FAO) in 2001. Probiotics are defined as ‘Live microorganisms which, when administered in adequate amounts, confer a health benefit on the host” [1,2]. A fundamental requirement for probiotic efficacy is their ability to survive the harsh conditions of the gastrointestinal tract [3,4], particularly the acidic environment of the stomach and the presence of bile salts [5]. Additional characteristics include adhesion to mucosal and epithelial surfaces [6,7], antimicrobial resistance in specific contexts, anti-mutagenic properties, and potential anticancer activities. Notably, probiotics have been associated with immunomodulatory effects, such as the regulation of inflammatory cytokines, stimulation of phagocytic activity, and modulation of both innate and adaptive immune responses [8].

The clinical interest in probiotics has expanded significantly in recent years, driven by the increasing global concern over antibiotic resistance, the demand for more targeted and host-friendly therapies, and the growing understanding of the gut microbiota’s role in human health [9]. One of the most promising applications of probiotics lies in their use as delivery platforms for therapeutic molecules. Through genetic engineering, probiotic strains can be modified to express heterologous genes encoding therapeutic peptides or proteins [10], opening new possibilities in the treatment and prevention of infectious diseases, inflammation, and even cancer [11].

However, several limitations hinder their broader therapeutic application. A significant obstacle is the lack of specific regulatory frameworks for genetically modified probiotics, which prevents their official registration as genetically engineered organisms in many countries [12]. Unlike conventional probiotics used in foods or supplements, Live Biotherapeutic Products (LBPs) are defined by the U.S. Food and Drug Administration (FDA) as biological products that contain live organisms applicable to the prevention, treatment, or cure of a disease but are not vaccines and do not fall under traditional dietary categories. This distinction creates regulatory uncertainty that will be discussed further in subsequent sections [13].

Moreover, challenges persist in the early development stages of LBPs, including ensuring safety, efficacy, and manufacturing quality. In contrast to conventional pharmaceutical products, LBPs currently lack standardized preclinical and clinical guidelines, as well as robust post-marketing surveillance systems [13,14,15]. The absence of established quality controls and biomarkers for efficacy remains a significant hurdle for their widespread clinical adoption. Additionally, safety concerns must be carefully evaluated, including the potential for horizontal gene transfer, unintentional immune activation, or disruption of host microbiota composition.

Physiological barriers also limit the effectiveness of LBPs, as these microorganisms must resist degradation by digestive enzymes and bile fluids to reach their target site [16]. Oral vaccines are considered one of the most promising applications of live biotherapeutic products (LBPs). However, they encounter intrinsic limitations associated with the tolerogenic environment of the gastrointestinal tract, which can attenuate antigen-specific immune activation and reduce overall vaccine efficacy [17]. To generate a protective immune response, these systems must cross the mucosal barrier, activate antigen-presenting cells (APCs), and ensure effective delivery of the antigen to the appropriate intestinal immune compartments [18].

Among the LBPs, *Lactococcus lactis*, a member of the lactic acid bacteria group, has emerged as a leading candidate. This bacterium has demonstrated considerable potential in oral vaccine development, particularly through modifications that enable the production of immunogenic proteins capable of eliciting measurable immune responses [15]. In addition to *L. lactis*, various *Lactobacillus* species have also been explored extensively as live vectors. Both microorganisms have a long history of safe consumption in fermented foods and are classified as Generally Recognized As Safe (GRAS), making them attractive alternatives to attenuated pathogens for antigen delivery at mucosal surfaces [19]. *Lactobacillus* spp. Often, they persist longer in the gastrointestinal and mucosal environment, eliciting strong local and systemic immune responses due to prolonged residence and intrinsic probiotic properties that facilitate host immune interaction. In contrast, *L. lactis* is considered the model LAB vector because of its well-established genetic tools, short residence time in the gut, and versatility for controlled antigen expression, minimizing risks of prolonged colonization and tolerance [20,21]. This review aims to summarize the most recent advancements in the use of genetically modified microorganisms, with a particular focus on *L. lactis* as a platform for vaccine delivery. We will examine both experimental and clinical research, highlighting opportunities, technological innovations, and regulatory limitations that define the current landscape and future potential of these live biotherapeutic systems.

## 2. Advantages of *Lactococcus lactis* for Its Application in LBPs

*Lactococcus lactis* is a non-pathogenic, Gram-positive bacterium that has been classified as “Generally Recognized As Safe” (GRAS) by the United States Food and Drug Administration (FDA). Traditionally used in the food industry to produce dairy products, fermented vegetables, and other fermented foods, *L. lactis* has long been valued for its metabolic simplicity, lack of endotoxins, and extensive history of safe human consumption [22].

The complete genome sequencing of various *L. lactis* strains [23,24] has facilitated the development of advanced genetic engineering tools, enabling the use of this bacterium as a vehicle for mucosal delivery of bioactive proteins [25]. Three major subspecies have been identified: *L. lactis* subsp. *lactis*, subsp. *cremoris*, and subsp. *hordniae*. Among these, *L. lactis* subsp. *cremoris* MG1363 has become the reference strain for genetic manipulation. This derivative of the ancestral NCDO712 strain (formerly classified as *Streptococcus lactis*) is plasmid-free, lacks phages, and is devoid of extracellular proteases, making it particularly suitable for laboratory applications [26,27]. In this line, MG1363 has been modified to remove the pLP712 plasmid, which encodes the lac operon and proteases required for casein degradation. As a result, this strain is incapable of growing in milk, which limits its survival and propagation outside controlled laboratory environments [27]. This biosafety feature, along with its genetic tractability, makes *L. lactis* an attractive chassis for synthetic biology applications.

Although *L. lactis* does not naturally colonize the gastrointestinal tract of humans or animals, it can transiently survive after oral administration, allowing short-term interaction with intestinal mucosal surfaces. In murine models (BALB/c), viable *L. lactis* cells have been detected in the small intestine up to 24 h after a single oral dose of 5 × 10^10^ CFU, with gradual clearance thereafter [28]. Similarly, in human volunteers, the strain MG1363 was detected in ileal effluents up to four hours after ingestion of 3 × 10^7^ CFU, confirming its transient persistence and low colonization potential [29]. This limited residence time is advantageous for biosafety, as it minimizes long-term colonization while permitting sufficient antigen release and interaction with gut-associated lymphoid tissues (GALT).

Beyond its favorable safety profile, *L. lactis* displays intrinsic immunomodulatory properties relevant to its use as a live vaccine vector. In vitro studies using human intestinal epithelial cell lines (HT-29 and Caco-2) have demonstrated that *L. lactis* stimulates the production of chemokines, such as IL-8, thereby promoting the recruitment of immune cells to mucosal sites [30]. In murine models, recombinant *L. lactis* strains expressing immunoregulatory molecules (e.g., IL-10) [31] or pathogen-derived antigens from *Helicobacter pylori* [32], HPV-16 [33], and *Listeria monocytogenes* [34] elicited antigen-specific immune responses, cytokine polarization, and protection against infection or tumor challenge. Together, these findings support the potential of *L. lactis* not only as a safe delivery vehicle but also as a biologically active adjuvant capable of shaping mucosal and systemic immune responses.

In addition, the favorable safety profile of *L. lactis* exhibits immunomodulatory properties such as an adjuvant by inducing chemokine expression both in vivo and in vitro. In addition, it can promote the maturation of bone marrow-derived dendritic cells [35,36]. These characteristics have positioned *L. lactis* as a promising platform for the development of mucosal vaccines, particularly for delivering antigens from viral, bacterial, and parasitic pathogens.

## 3. Conventional Methods and Challenges for Antigen Expression and Secretion in *Lactococcus lactis*

The unique combination of safety, transient persistence, and immunomodulatory potential has established *L. lactis* as one of the most versatile bacterial chassis for live biotherapeutic and vaccine applications [22]. However, the success of this platform largely depends on the efficiency and regulation of heterologous gene expression. A range of expression systems has been developed to optimize antigen synthesis, secretion, and surface display in *L. lactis*, each offering distinct levels of control and applicability for mucosal vaccine design. The following section summarizes the main inducible and physiological systems currently employed for antigen expression in *L. lactis*, highlighting their mechanisms of regulation, strengths, and limitations (Table 1).

### 3.1. Nisin-Controlled Gene Expression (NICE)

One of the most widely adopted platforms for heterologous gene expression in *L. lactis* is the Nisin-Controlled Gene Expression (NICE) system [37]. Initially developed by de Ruyter and collaborators [38]. This system allows for tightly regulated and dose-dependent induction of gene expression in response to nisin, an antibiotic approved by the U.S. Food and Drug Administration (FDA) in 1988 and commonly used as a food preservative [39].

**Figure 1:**
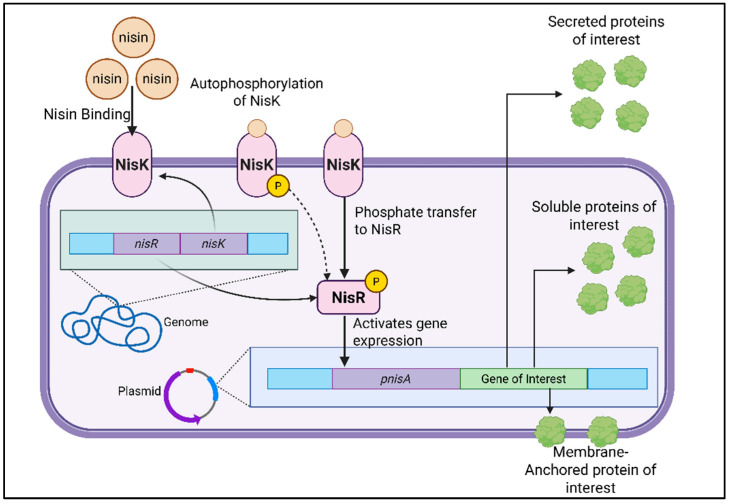
Upon external addition of nisin, it binds to the membrane-bound histidine kinase NisK, triggering its autophosphorylation. NisK then transfers the phosphate group to the response regulator NisR. Phosphorylated NisR activates transcription from the PnisA promoter, leading to the expression of the gene of interest. The expressed protein can then be directed for secretion, remain soluble in the cytoplasm, or be anchored to the bacterial membrane, depending on its signal peptide or fusion tags. Based on [36] and created in BioRender. Soto, J. (2025) https://BioRender.com/pibhxg1.

The NICE system operates through a two-component signal transduction mechanism, comprising the membrane-bound histidine kinase NisK and the cytoplasmic response regulator NisR (Figure 1) [37]. When nisin is added to the culture medium, it binds to NisK, initiating autophosphorylation. NisK then transfers the phosphate group to NisR, activating it. Phosphorylated NisR subsequently induces transcription from nisin-responsive promoters, primarily the PnisA promoter. Notably, the promoter driving the expression of the *nisK* and *nisR* genes remains constitutively active. It is not regulated by nisin, thereby ensuring basal levels of the regulatory proteins regardless of external stimuli [40].

Initially, the NICE system was implemented in *L. lactis* NZ9700; this strain is a transconjugant from a mating between the nisin-A-producing strain NIZO R5 and the plasmid-free strain MG1614 and contains a single copy of the nisin-sucrose transposon *Tn5276* at the same chromosomal site as in *L. lactis T165.1* and *T165.5* [41]. The system was further optimized in *L. lactis* subsp. *cremoris* MG1363 by Kuipers and collaborators, who engineered a strain (NZ9000) by inserting *nisK*, *nisR*, the 3′ end of *nisP*, and the 5′ end of *nisF* into the chromosome, replacing parts of the *pepN* and *napC* genes [42]. This enhanced strain became the standard for NICE-based expression. In *L. lactis* NZ9000, gene induction is achieved by adding subinhibitory concentrations of nisin, typically ranging from 0.1 to 10 ng/mL [43].

### 3.2. Xylose Inducible Expression System (XIES)

In addition to the widely used NICE system, *L. lactis* has also been engineered to utilize the Xylose-Inducible Expression System (XIES), which offers a tightly regulated alternative for heterologous gene expression. This system is based on the PxylT promoter, identified in the xylose utilization operon of *L. lactis* subsp. *lactis* NCDO2118, which controls transcription of the *xylT* gene encoding a xylose permease [44]

Functional characterization of PxylT demonstrated that it contains a conserved catabolite-responsive element (cre) [45]. In the presence of xylose, the transcriptional activator XylR binds to PxylT and strongly induces transcription—up to 10,000-fold during mid-exponential growth (OD_600_ ≈ 0.4). This promoter can be repeatedly switched on by the addition of xylose and off by washing and resuspending cells in glucose-containing medium, providing precise, reversible, and food-grade control over gene expression [44].

With these findings, Miyoshi and collaborators developed a complete xylose-inducible expression system combining PxylT with the ribosome-binding site and secretion signal peptide (SP) of the Usp45 protein [46], fused to the *Staphylococcus aureus* nuclease gene (*nuc*) as a reporter [47]. This configuration allowed targeted expression of heterologous proteins either in the cytoplasm or secreted into the extracellular medium, demonstrating efficient and controllable protein production in the vegetable isolate *L. lactis* NCDO2118.

Compared with the nisin-inducible NICE system, XIES presents several advantages: it does not require antibiotic or peptide inducers, making it safer and more cost-effective; and its regulatory logic directly couples gene expression to the carbon source, which is advantageous for food-grade applications [48]. Nonetheless, its practical use is restricted to strains that can metabolize xylose; *L. lactis* NZ9000 lacks the xylose utilization pathway [24]. Comparative analyses revealed that the cytoplasmic and secreted production of the reporter nuclease (Nuc) in *L. lactis* NCDO2118 was approximately tenfold higher under nisin induction than under xylose induction during exponential growth, although both systems reached similar expression levels in the stationary phase [44], which can affect the timing of protein synthesis. Despite these limitations, the XIES provides a reliable, tunable, and biosafe alternative for regulated protein production in *L. lactis*, expanding its applicability in mucosal vaccine and biotherapeutic development.

### 3.3. Zinc-Induced Systems

The PZn–zitR system is a zinc-repressible expression system described by Llull and collaborators [49] derived from the zit operon of *L. lactis*, which encodes the high-affinity zinc uptake ABC transporter ZitSPQ and its regulator ZitR. The regulatory mechanism is based on the ZitR repressor, a MarR-family metalloregulator that binds to the PZn promoter in the presence of excess zinc, thereby blocking transcription of downstream genes. When extracellular zinc becomes limited, ZitR dissociates from the promoter, allowing transcription to proceed. Consequently, the system is activated under conditions of zinc depletion, either naturally during bacterial growth or through chelation with agents such as EDTA. Thus, the system is activated by zinc depletion and can be induced through natural metal starvation or by adding chelating agents such as EDTA. This system provides tight regulation, gradual induction during bacterial growth, and is compatible with food-grade applications, as it does not rely on synthetic inducers like nisin. However, its limitations include the need for precise control of metal concentrations, possible interference with cell metabolism due to chelation, and lower maximal expression levels compared to the nisin-controlled expression (NICE) system. Despite these constraints, PZn–zitR remains a valuable tool for the controlled production of heterologous proteins in *L. lactis*, especially in mucosal or physiological environments where environmental cues naturally modulate gene expression [49].

In contrast, Zirex, designed by Mu and collaborators [50], introduced the SczA–PczcD regulatory module from *Streptococcus pneumoniae* into *L. lactis*, creating the first zinc-inducible expression system for this species. In Zirex, the transcriptional activator SczA binds to two specific motifs within the PczcD promoter to repress transcription in the absence of zinc and activate it in response to Zn^2+^ supplementation [50]. Experimental analyses in *L. lactis* NZ9000 showed that induction with 0.3–0.7 mM ZnSO_4_ during the exponential phase produced strong green fluorescent protein (GFP) expression, reaching approximately 80% of the levels achieved with the nisin-inducible PnisA promoter, while maintaining negligible basal expression under uninduced conditions. Moreover, Zirex exhibited low toxicity even at 0.5 mM Zn^2+^ and allowed precise, dose-dependent induction between 0 and 0.3 mM Zn^2+^. A key advantage of Zirex is its compatibility with other inducible systems: the designers demonstrated a dual-promoter configuration, combining PczcD and PnisA to independently express GFP and mCherry, achieving simultaneous expression with only minor signal interference (~10–20%). These properties make Zirex particularly attractive for the controlled expression of metalloenzymes, zinc-binding antigens, and antibiotic biosynthetic enzymes, where zinc serves both as inducer and cofactor. Although careful optimization of Zn^2+^ concentrations is required to prevent metal stress, Zirex offers a robust, tunable, and food-grade alternative for controlled heterologous protein production in *L. lactis*, expanding the available molecular toolbox for mucosal vaccine and biotherapeutic development [50].

More recently, Xu and collaborators reported the ZICE (Zn^2+^-Controlled Expression) system [51], derived from *Streptococcus thermophilus*, which was successfully applied in *L. lactis* NZ9000 [51]. The system is based on the sczAst–PczcDst regulatory module, homologous to that of *S. pneumoniae*, and provides precise zinc-dependent activation. In *L. lactis* NZ9000, induction with 0.8 mM ZnSO_4_ at mid-exponential growth (OD_600_ ≈ 0.4) resulted in strong expression of GFP and secreted IL-10, reaching approximately 60% of the levels obtained with the NICE system. Notably, the ZICE platform showed no background expression, tight on/off control, and improved expression when yeast extract was removed from the medium, suggesting an interaction between nitrogen availability and metal ion regulation. The stability, low cost, and physiological relevance of zinc as an inducer make ZICE a promising tool for the development of food-grade and probiotic-based expression systems.

Each of the zinc-responsive expression systems developed for *Lactococcus* spp. presents distinctive strengths and constraints depending on the regulatory mechanism and intended application. The native PZn–zitR system offers exceptional regulatory tightness and food-grade compatibility, as it relies exclusively on intrinsic metabolic control without requiring exogenous inducers. However, its activation depends on zinc depletion, which can be difficult to maintain under standard culture conditions and may alter cell physiology when strong chelating agents are used. The Zirex system, in contrast, provides high-level, zinc-inducible expression that reaches up to 80% of the activity of the nisin-controlled promoter while preserving minimal basal leakage. Its dose-dependent and reversible behavior makes it ideal for fine-tuned laboratory applications. However, the use of regulatory elements derived from *S. pneumoniae* may limit its classification as fully food-grade. The recently developed ZICE system represents a safe alternative, originating from *S. thermophilus*, a species with GRAS status. Although ZICE achieves slightly lower maximal expression (45–60% of NICE levels), it combines food-grade safety, low background activity, and simple induction through ZnSO_4_ supplementation.

### 3.4. Stress Induces Controlled Expression System (SICE)

The study by Benbouziane and collaborators designed the Stress-Inducible Controlled Expression (SICE) system in *L. lactis*, aiming to enhance the safety and physiological relevance of mucosal vaccine delivery [52]. This system relies on the *groESL* promoter, which is activated by host-related stress conditions such as heat, acidity, or bile salts [53,54], allowing in situ production and secretion of the antigen or therapeutic protein only during bacterial transit through the host. Using this approach, *L. lactis* strains expressing IL-10 or the HPV-16 E7 antigen successfully induced localized immune responses and tumor protection in murine models [52].

Recent studies further support the use of stress-responsive promoters as auto-inducible alternatives. A heat-shock promoter (Phsp) derived from *Enterococcus faecium* was characterized and shown to drive heterologous gene expression in both *L. lactis* and *Lactobacillus plantarum* under heat, salt, and pH stress conditions. Phsp activity is regulated by the class III stress response repressor CtsR, allowing low basal expression under non-stress conditions and strong induction upon exposure to industrially relevant stresses. This promoter was successfully incorporated into the shuttle vector pAR1801, enabling auto-inducible protein expression without the need for external inducers such as nisin, thereby reinforcing the potential of the stress-inducible system for safe, cost-effective, and physiologically relevant expression in LAB [55].

The main advantages of the Stress-Induced Controlled system lie in its auto-regulated expression, which eliminates the need for external inducers like nisin, and its localized activation within mucosal environments, thereby improving biosafety and simplifying vaccine administration. However, limitations include potential variability in expression levels due to fluctuating stress conditions and the episomal nature of the plasmid, which may lead to plasmid instability in the absence of selective pressure.

Overall, the SICE platform represents a promising strategy for mucosal immunization, combining controlled antigen release, host-responsive regulation, and the intrinsic safety of *L. lactis* as a non-pathogenic live vector for delivery [52,56].

### 3.5. Low-pH-Inducible Expression Systems

One of the systems used in the expression of recombinant protein in *L. lactis* is the P170 based on a chromosomal promoter from *L. lactis* MG1363 that is auto-induced in response to lactic acid accumulation and low pH conditions associated with the transition to the stationary phase [57,58]. Induction of the P170 promoter is mediated by the transcriptional regulator RcfB, a member of the Crp/Fnr family, which interacts with a conserved upstream sequence known as the ACiD box [59,60]. This regulatory mechanism is specifically responsive to lactate, whether produced endogenously during homofermentative growth or added exogenously, and correlates with growth inhibition and adaptation to acid stress [58,59]. Unlike chemically inducible systems, P170 does not require external inducers, thereby simplifying process control and facilitating scalability for industrial fermentations [60].

The performance of the P170 expression system was evaluated in *L. lactis* strains engineered for hyaluronic acid production and directly compared with the nisin-inducible NICE system. Recombinant strains SJR3 (hasABC) and SJR6 (hasABD) served as NICE-based references, while equivalent gene combinations were expressed under the control of the auto-inducible P170 promoter. In both batch and fed-batch bioreactor cultures, P170-driven expression consistently outperformed the NICE system. Under batch conditions, P170-based strains achieved up to ~50% higher hyaluronic acid titers and significantly increased molecular weight, with HA polymers exceeding 2.5 MDa at higher initial glucose concentrations. In contrast, NICE-based strains produced HA of lower molecular weight under comparable conditions. Similar trends were observed in fed-batch experiments, where P170 strains reached 40–45% higher HA concentrations and consistently higher molecular weights. These results suggest that auto-induction of the P170 promoter during the late-exponential growth phase enables a more favorable temporal separation between biomass formation and HA biosynthesis, resembling the native regulation of the has operon in *Streptococcus zooepidemicus* and likely reducing competition with central carbon metabolism and cell wall precursor synthesis [61]. Another interesting study that uses this system consists of the one by Cho and collaborators. The expression of the HPV16 L1 antigen under the p170 promoter in *L. lactis* MG1363 elicited both systemic and mucosal immune responses in mice, with antigen localization influencing the immune profile: intracellular expression favored IgA responses, whereas secreted antigen enhanced serum IgG levels [62].

The P170 expression system offers several advantages, including autoinduction driven by lactate accumulation, elimination of external inducers, and robust performance in batch and fed-batch fermentations [61], which together simplify process control and enhance scalability. Its induction during late-exponential growth enables improved temporal separation between biomass formation and product synthesis, benefiting metabolically demanding pathways. However, P170 activity is tightly coupled to acid stress and growth inhibition, which may limit maximal biomass and require careful process optimization [60,63].

### 3.6. Agmatine-Controlled Expression System

Another alternative inducible expression strategy in *L. lactis* is the Agmatine-Controlled Expression (ACE) system, which enables tightly regulated heterologous gene expression in response to extracellular agmatine. This system was developed based on the regulatory elements of the agmatine deiminase (AGDI) gene cluster from *L. lactis* subsp. *cremoris* CECT 8666, which is naturally involved in agmatine catabolism and putrescine biosynthesis [64].

ACE relies on the membrane-associated transcriptional regulator AguR, which is constitutively expressed and activates the PaguB promoter upon sensing extracellular agmatine. Unlike the NICE system, ACE operates through a single-component regulatory mechanism, simplifying its genetic architecture. To prevent carbon catabolite repression mediated by Catabolyte control protein A (CcpA), a point mutation was introduced into the cre site of PaguB, allowing efficient induction in glucose-containing media [65].

The resulting pACE vector was validated in *L. lactis* NZ9000 using reporter and enzymatic proteins, demonstrating strict agmatine-dependent induction with no detectable background expression. Maximal expression was achieved at 0,5 mM agmatine concentrations, and recombinant proteins were predominantly recovered in the soluble fraction. Comparative analyses indicated that expression levels obtained with ACE were comparable to, or higher than, those achieved with the NICE system under similar conditions. Although high agmatine concentrations slightly affected bacterial growth, final cell densities remained suitable for industrial-scale protein production [65].

In summary, the ACE system represents a robust, tightly regulated, and cost-effective alternative for inducible gene expression in *L. lactis*. Its single-component regulatory mechanism, lack of basal expression, and compatibility with strains lacking nisK/nisR expand the genetic toolbox available for this species. As such, the ACE system complements existing platforms, such as NICE and XIES, and constitutes a valuable option for the controlled production of recombinant proteins, including antigens and biotherapeutics intended for mucosal delivery, when *L. lactis* NZ9000 is not used.

### 3.7. Chloride-Inducible Gene Expression

An alternative inducible platform for heterologous gene expression in *L. lactis* is the chloride-inducible expression system, which is derived from regulatory elements of the *gad* operon [66,67]. This system was originally described by Sanders and collaborators through the identification of a sodium chloride-inducible promoter using single-copy chromosomal lacZ fusions in *L. lactis* MG1363 [66]. The use of chloride ions as an inducing signal is particularly attractive for dairy and food-related applications, as salt is a natural and widely accepted component of fermentation processes in adequate amounts [67].

This system relies on the transcriptional activator GadR and the chloride-responsive promoter Pgad, which regulate expression of the gadCB operon involved in acid stress response. GadR is constitutively expressed and activates transcription from *Pgad* in response to elevated extracellular chloride levels. Promoter activity increases in a dose-dependent manner, with maximal induction typically observed at NaCl concentrations of approximately 0.3–0.5 M, while basal expression in the absence of chloride remains negligible [68].

The gadR–Pgad regulatory module was subsequently adapted as a controllable expression system in *L. lactis* MG1363, demonstrating tight regulation and high induction ratios exceeding 1000-fold upon supplementation with NaCl. This platform was successfully used to control the expression of reporter proteins and biologically active enzymes, including proteins whose constitutive expression would be detrimental to bacterial viability, highlighting its stringent on/off behavior [66]. The chloride-inducible system has been primarily validated in *L. lactis* MG1363 and related strains, such as LL108 (Cm^r^ repA^+^) and LL302 (repA^+^), in the same study, with gene induction achieved by supplementing the culture medium with food-grade sodium chloride. This suggests that it could also be possible to use this system in *L. lactis* NZ9000. Although the relatively high salt concentrations required for maximal induction may affect bacterial growth and limit in vivo applications, the simplicity, low cost, and food-grade nature of the inducer make this system a valuable alternative to peptide-based platforms such as NICE, expanding the molecular toolbox for regulated gene expression in *L. lactis*.

### 3.8. Phosphate Starvation-Inducible Expression System

The phosphate starvation-inducible expression system in *L. lactis* is based on transcriptional regulation mediated by the Pho regulon, as described in other bacteria such as *Escherichia coli* and *Bacillus subtilis* [69]. On *L. lactis*, Sirén and collaborators developed a gene expression system driven by the *pstF* promoter, which is associated with a high-affinity phosphate transport operon [70]. Under phosphate-replete conditions, promoter activity remains tightly repressed; however, phosphate limitation triggers activation through a conserved two-component regulatory mechanism involving a membrane-associated histidine kinase and a cognate response regulator, leading to strong transcriptional induction [71]. This regulatory logic enables growth-phase-dependent, auto-inducible expression without the need for exogenous inducers [70].

The performance of the system was evaluated using both intracellular and secreted model proteins in *L. lactis*. Under phosphate-depleted conditions, the pstF promoter supported robust expression of β-galactosidase and efficient secretion of α-amylase, with minimal basal activity observed in phosphate-rich media. Induction occurred when inorganic phosphate concentrations dropped below a defined threshold and could be rapidly reversed by phosphate supplementation, demonstrating tight and reversible control. Importantly, expression levels achieved under phosphate starvation were comparable to those obtained with NICE system under standard induction conditions. Moreover, the system remained functional at bioreactor scale, where induction was driven solely by natural phosphate consumption during growth, without the need for medium manipulation or inducer addition [70].

From an applied perspective, the phosphate starvation-inducible system offers several advantages, including its inducer-free nature, food-grade compatibility, low basal expression, and suitability for large-scale fermentation processes. The absence of a requirement for heterologous regulatory genes further simplifies strain construction and enhances versatility across *L. lactis* backgrounds. However, the system also presents limitations, notably reduced temporal precision compared to inducible systems and dependence on nutrient depletion, which may vary depending on the medium composition or growth conditions. The authors mentioned that the phosphate levels in the bioreactor fluctuated during the experiment which can complicate the analysis and development for mucosal vaccines. Despite these disadvantages, phosphate starvation responsive expression represents an interest and physiologically relevant alternative for controlled heterologous protein production in *L. lactis*, particularly for industrial, food-grade, mucosal vaccines and applications where external induction is impractical or when it is necessary to avoid the use of antibiotic resistance gene.

### 3.9. Strategies to Enhance Secretion of Heterologous Antigens in L. lactis

A major challenge in the development of *L. lactis*-based vaccines is achieving efficient secretion of heterologous antigenic proteins. Low secretion yields can limit antigen availability at mucosal sites, reducing both immunogenicity and overall vaccine efficacy [72,73]. Several strategies have been developed to optimize secretion in *L. lactis*, with a primary focus on signal peptide selection, peptide engineering, and protein fusion approaches.

One of the most widely employed strategies involves the use of the native signal peptide (SP) of Usp45, which directs proteins to the Sec-dependent secretion pathway and has been shown to outperform heterologous signal peptides in *L. lactis* [74]. The insertion of short synthetic pro-peptides, such as LEISSTCDA, between the signal peptide and the mature protein can significantly enhance secretion efficiency and protein yield by improving translocation and folding [75].

Beyond native SPs, heterologous signal peptides can be optimized to surpass Usp45 performance. An interesting study demonstrated that heterologous signal peptides can outperform Usp45 when carefully optimized. The SPK1 from *Pediococcus pentosaceus* was modified through targeted mutations in its N-, H-, and C-domains, and tested for the secretion of *Staphylococcus aureus* nuclease (NUC) in *L. lactis*. Several SPK1 variants enhanced secretion efficiency, with the best mutant, SPKM19, achieving up to a 1.4-fold increase and a 1.7-fold improvement in secretion activity yield compared to the wild-type SPK1. Notably, mutations in the cleavage site C-domain improved secretion, whereas changes in the H-domain were detrimental. These findings highlight the potential of developing heterologous SPs with higher efficacy than Usp45 for enhanced antigen secretion and mucosal delivery applications in *L. lactis* [76].

Additional approaches to improving secretion include fusion to carrier proteins or stabilizing domains, which protect labile antigens from cytoplasmic degradation and facilitate export [72], as well as co-expression with chaperone-like factors, which can protect labile antigens from cytoplasmic degradation and facilitate export. For example, co-expression of the *Bacillus subtilis* chaperone-like protein PrsA significantly increased total and secreted yields of heterologous proteins in *L. lactis* [77]. Similarly, the development of a thioredoxin (TrxA) fusion system in *L. lactis*, based on strategies previously applied in *E. coli*, enabled high-level production of soluble heterologous proteins that were previously insoluble or not expressed, while preserving protein integrity and allowing efficient purification and cleavage of the fusion TrxA using enterokinase [78].

### 3.10. Surface Display of Antigens in L. lactis

In addition to secretion, heterologous antigens can be delivered via cell-surface display in *L. lactis*. Several anchoring strategies have been developed, including fusion to cell-wall motifs such as the *Streptococcus pyogenes* M6 protein (CWAM6) [79], the H and W domain of PrtB from *Lactobacillus delburueckii* subsp. *bulgaricus* [80], LysM domains [81], among other strategies; these are summarized in the study of Michon and collaborators [82].

Surface display provides several advantages over secretion, including prolonged antigen exposure at mucosal sites, enhanced recognition by antigen-presenting cells, and reduced degradation compared to secreted proteins [83]. However, display efficiency may be limited by antigen size [84] or folding constraints [85], potentially reducing immunogenicity in some cases. Another disadvantage is the capability to express complex eukaryotic antigens that require post-translational modifications to exhibit activity [86].

Notably, some display strategies can be implemented without genetic modification of the host, enhancing biosafety and regulatory acceptance. Examples include non-covalent or covalent anchoring of antigens to the bacterial surface after expression. Ramasamy and collaborators demonstrated that the *Plasmodium falciparum* merozoite protein MSA2, non-covalently bound to non-genetically modified *L. lactis* GEM particles, elicited systemic and mucosal antibody responses comparable to those obtained with covalently anchored recombinant *L. lactis*, highlighting the potential of non-GMO platforms for oral vaccination [87]. Similarly, Varma and collaborators displayed Enterovirus 71 VP1 antigens on *L. lactis* by docking purified fusion proteins, successfully inducing immune responses in mice [88]. Ribelles and collaborators reported that non-GMO LAB displaying HPV-16 E7 on *L. lactis* and *Lactobacillus casei* achieved effective mucosal immunization in murine models [89]. Kalyanasundram and collaborators extended this concept to glycosylated mammalian antigens, demonstrating the successful anchoring of Tyrosinase-related protein 2 to L. lactis cell walls, which opens new avenues for therapeutic vaccine development [81]. Collectively, these studies illustrate that non-genetically modified surface display approaches offer safe, versatile, and effective antigen presentation strategies suitable for clinical translation and food-grade applications. Such non-GMO approaches are particularly attractive for the development of safety mucosal vaccines and food-grade applications, while still providing effective antigen presentation.

**Table 1:** Advantages and disadvantages of the main antigen expression methods for *L. lactis*.

System	Inductor	Advantages	Disadvantages	Refs.
NICE	Nisin	- Tight, dose-dependent control - High expression levels - Well-characterized and widely used - Suitable for secretion or surface display	- Requires addition of nisin (non-food-grade inducer in some contexts) - Possible background expression - Limited in complex media containing nisin inhibitors	[37,38,40,41]
XIES	Xylose	- Food-grade, no antibiotics or peptides - Cost-effective inducer - Direct link between metabolism and expression	- Requires strains able to metabolize xylose - Slower induction during exponential growth - Lower maximal expression than NICE	[44,90]
Zinc Repressible System	Zinc depletion or chelation (EDTA)	- Tight repression and gradual induction - Food-grade and environmentally responsive - Ideal for metal-dependent regulation studies	- Requires precise control of Zn^2+^ concentration - Chelation can disturb cell physiology - Lower expression yield than NICE	[49]
Zirex	Zn^2+^	- Strong induction (~80% of NICE) - Very low basal expression - Dual-promoter compatibility (can combine with NICE) - Suitable for metalloenzyme production	- Requires fine-tuning of zinc concentration - May vary between strains - Still less standardized than NICE	[50]
ZICE	Zn^2+^	- Completely food grade (GRAS) - No basal expression	- Lower expression than NICE (45–60%)	[51]
SICE	host-related stress (heat, acidity, bile salts) through the groESL promoter	- Auto-regulated and self-limiting - Activation during host transit (in situ expression) - No external inducer required - High biosafety potential	- Variable induction due to stress conditions - Episomal plasmid may be unstable	[52]
P170	Lactate accumulation/low pH	- Auto-induced - Robust in batch/fed-batch	- Activation is linked to acid stress and growth limitation	[57,58,59,60]
ACE	Agmatite	- No basal expression - Works in strains lacking NisK/NisR	- High levels of agmatite can affect growth	[65]
Chloride-inducible	NaCl	- Low cost - Food grade inducer	- High salt may affect growth	[66,68]
Phosphate starvation	Phosphate depletion	- No inducer needed - Low basal expression	- Less temporal precision - Phosphate fluctuations complicate control	[70]

## 4. Routes of Administration

The mucosal immune system is organized into functionally interconnected compartments called mucosa-associated lymphoid tissue (MALT), which includes the gut-associated lymphoid tissue (GALT), nasal-associated lymphoid tissue (NALT), bronchial-associated lymphoid tissue (BALT), skin-associated lymphoid tissues (SALTs), and genital organ-associated lymphoid tissues (GOALTs), among others [91]. Antigen exposure at mucosal sites triggers distinct immune pathways, influencing both the magnitude and quality of the immune response. Selecting the administration route for vaccines based on *L. lactis* is essential to induce the desired immune response, toward immune tolerance for the treatment of autoimmune and allergic diseases or toward robust protective immunity against pathogens [92]. Typically, the strongest immune response is elicited at the site of immunization and in anatomically adjacent mucosal sites [93].

### 4.1. Oral Administration

Oral administration is the most common and natural route for delivering *L. lactis*, especially in vaccine and immunomodulatory therapies [94]. This route is convenient, non-invasive, and advantageous for stimulating mucosal immunity within the gastrointestinal tract, primarily through the gut-associated lymphoid tissue (GALT) [95]. It can also induce a substantial local secretory IgA (SIgA) antibody response in the proximal part of the small intestine, the ascending colon, the stomach, and in the mammary and salivary glands [96]. Moreover, oral delivery enhances patient compliance and is suitable for both preventive and therapeutic applications [17,18]. However, a critical limitation of oral administration is the induction of oral tolerance, an immunological mechanism that prevents excessive inflammatory responses against dietary antigens and the resident microbiota [97]. Oral tolerance is characterized by local and systemic immune unresponsiveness following antigen exposure via the oral route [98]. This oral tolerance is mainly mediated by CD4^+^ regulatory T cells (Tregs) expressing the transcription factor FoxP3 [99]. These cells suppress immune responses through multiple mechanisms, including the production of inhibitory cytokines such as transforming growth factor-beta (TGF-β), interleukin IL-10, and IL-35. Also, they can release cytotoxic enzymes like granzyme and perforin, consume and degrade ATP and IL-2, and express inhibitory surface molecules such as LAG-3 and CTLA-4 [100].

In the mucosal immunity, IL-10, TGF-β, and CTLA-4 play fundamental roles in maintaining homeostasis, preventing excessive inflammation, and preserving epithelial barrier integrity [101,102,103]. Mucosal FoxP3^+^ Tregs frequently express IL-10 [104], which suppresses activation of myeloid cells [105], gamma delta T cells [106], and conventional CD4^+^ T cells [107,108]. STAT3 regulates IL-10 expression, crucial for autocrine activation of Tregs and suppression of Th17 responses [109,110]. TGF-β is essential for Treg suppressive function by enhancing effector activity through the SMAD signaling pathway [111,112]. CTLA-4, an inhibitory receptor constitutively expressed under FoxP3 control [113,114], competes with CD28 for CD80/CD86 ligands on dendritic cells (DCs), modulating immune activation [115,116]. Moreover, CTLA-4 expression in Tregs is induced by the microbiota, reinforcing its role in mucosal immune regulation [117]. Tregs also suppress immune responses via competitive and passive mechanisms, including aggregation around DCs mediated by high LFA-1 expression [118] and efficient sequestration of IL-2 from activated T cells due to high IL-2 receptor expression (CD25 and CD122), without producing IL-2 themselves, reinforced by FoxP3-mediated transcriptional repression of IL-2 [119,120,121,122].

In addition to immunological challenges, oral administration exposes *L. lactis* to harsh physical and chemical barriers, including gastric acidity, digestive enzymes, and competition from resident microbiota, all of which can reduce viability and antigen delivery efficiency [16]. Variability in effective dose delivery also presents challenges to consistent therapeutic outcomes.

### 4.2. Nasal Administration

Nasal administration offers an alternative route for delivering *L. lactis* to stimulate mucosal immunity in the respiratory tract. This route bypasses gastrointestinal barriers and can induce both local mucosal and systemic immune responses [123]. The nasal-associated lymphoid tissue (NALT) acts as the primary inductive site for immune activation in this route, containing microfold (M) cells, dendritic cells (DCs), and organized lymphoid follicles that facilitate efficient antigen sampling and presentation to naive T and B lymphocytes [91]. Following antigen uptake through microfold (M) cells and presentation by mucosal dendritic cells, B cell activation and class switching to IgA are promoted through cytokines such as IL-5 and IL-6, leading to robust secretory IgA (SIgA) production in the upper respiratory tract [124,125]. In addition, activated lymphocytes from NALT migrate to distant effector sites, including the oral, pulmonary, and genital mucosa, through the common mucosal immune system, generating coordinated humoral and cellular protection [126,127].

Nasal immunization can induce Th1/Th2-balanced or Th17-type immune responses depending on the antigen, adjuvant, or bacterial vector employed, resulting in effective cytotoxic T lymphocyte (CTL) responses and long-term memory formation [128,129,130]. Compared with oral administration, the nasal route avoids enzymatic degradation and first-pass tolerance in the gastrointestinal tract, providing a more efficient and rapid activation of mucosal and systemic immunity [131]. However, limitations include mucociliary clearance, restricted dosing volume, and antigen instability within the nasal environment, which may compromise bacterial viability and immunogenicity. Strategies such as mucoadhesive formulations, nanoparticle encapsulation, or co-delivery with mucosal adjuvants are under development to improve vaccine retention and efficacy via this route [125].

### 4.3. Other Routes of Administration

Rectal immunization induces a pronounced local SIgA response in the rectum and sigmoid colon, a moderate response in the descending colon, and minimal or no response in the proximal colon or small intestine. In contrast, nasal or tonsillar immunization in humans primarily triggers antibody production in the upper respiratory mucosa and its associated secretions, without activating immune responses in the intestinal tract [132]. Interestingly, nasal administration has also been shown to generate strong IgA and IgG responses in the human cervicovaginal mucosa, achieving levels comparable to those seen with direct vaginal immunization. Although the capacity of nasal immunization to prime CD4^+^ and CD8^+^ T cells in the genital tract remains incompletely characterized, evidence from murine models suggests that vaginal vaccination is more effective in inducing strong genital tract T cell-mediated immunity [133]. Sublingual and topical applications have been explored in preclinical settings for *L. lactis*-based therapies. These methods aim to utilize mucosal immunity in different compartments or to support specific clinical applications; however, evidence remains limited, and additional research is needed to evaluate their feasibility and effectiveness [92].

## 5. Oral Vaccine Prototypes on *L. lactis*

Oral vaccines based on genetically modified *L. lactis* have shown promising results in preclinical models against a variety of infectious agents [134]. These models typically involve the expression of selected antigens chosen for their immunogenicity and role in pathogen virulence or protection, aiming to elicit both mucosal and systemic immune responses. Different vaccination schemes have been explored, often combining mucosal priming with systemic boosting to enhance immune memory and facilitate the effective homing of immune cells to mucosal tissues.

### 5.1. Viral Vaccines

Human Papilloma Virus (HPV) is a significant cause of cervical cancer worldwide [92]. Existing prophylactic vaccines, such as Gardasil and Cervarix, use virus-like particles (VLPs) to induce immunity. In contrast, Mohseni et al. employed *L. lactis* to deliver the E7 oncoprotein of HPV16, a key antigen involved in oncogenesis. This choice targets the therapeutic elimination of infected cells expressing E7. Their studies demonstrated increased E7-specific antibody titers and CD4^+^ T cell responses, showing promise as a therapeutic vaccine platform [135]. These outcomes were further validated in early-phase clinical trials [136], supporting the translational potential of this approach. A more detailed review was conducted by the same author [33]. These results contribute to reducing cervical cancer.

The absence of a protective prophylactic HIV vaccine has allowed for the exploration of different platforms to control the spread of this virus [137]. *L. lactis*-based oral vaccines have been developed that express HIV antigens, such as the Gag protein fused to the T3 pilus protein of *Streptococcus pyogenes*, thereby enhancing mucosal delivery [138]. This strategy elicited increases in Gag-specific IgG and IgA in serum, feces, and vaginal secretions, and activated dendritic cells in Peyer’s patches, although CD8^+^ T cell responses remained low. Another approach targeted the V2–V4 “loop” of the HIV envelope protein (Env), achieving systemic and mucosal IFN-γ responses with repeated oral dosing every two weeks [139]. Considering the importance of HIV in public health, a mucosal vaccine against this virus represents a valuable contribution to the development of a vaccine against this pathogen.

Influenza A virus subtypes H1N1 and H3N2 cause seasonal epidemics [140]. Oral vaccines using *L. lactis* have targeted hemaglutinin (HA), the major surface antigen. Lei and collaborators demonstrated that *L. lactis* expressing HA of the H5N1 subtype elicited significant IgG and mucosal IgA responses, conferring complete protection in mice against lethal viral challenge after multiple immunizations [141,142]. More recently, a recombinant strain expressing the conserved HA stalk domain fused to a bacterial anchoring protein demonstrated cross-protection against H5N1, H3N2, and H1N1 strains, highlighting the potential for developing a universal influenza vaccine [142].

Hepatitis B Virus (HBV) causes chronic liver disease, with existing vaccines available, but a continuous need for novel strategies. Early studies expressed the PreS region of the HBV surface antigen (HBsAg) in *L. lactis*, inducing intestinal IgA and serum IgG in mice [143]. Co-expression of IFN-γ as an adjuvant further enhanced the IgG response. More recent efforts include the expression of central HBsAg regions from different HBV genotypes; however, preclinical results remain pending [144].

The first *L. lactis*-based vaccine against the Severe Acute Respiratory Syndrome (SARS) utilized the nucleocapsid (N) protein fused to human GST, inducing specific serum IgG responses in mice [145]. Following the COVID-19 pandemic, *L. lactis* models expressing the receptor-binding domain (RBD) of the spike (S) protein or conserved S protein regions have been developed, aiming to stimulate protective mucosal and systemic immunity [146]. Another study utilized the highly conserved region (HCR) of the Spike S2 subunit, driven by the nisin-inducible pNZ8149 vector to express the antigen. Mice immunized through oral or intranasal routes exhibited significantly increased levels of anti-SARS-CoV-2 IgG and IgA, along with elevated CD4^+^ and CD8^+^ T-cell responses in lymphoid and intestinal tissues. Notably, the intranasal route elicited stronger humoral and cellular responses, consistent with the activation of nasal-associated lymphoid tissue (NALT) and enhanced mucosal immunity [147]. Importantly, the construct was generated under food-grade conditions, employing a lactose-based selection system rather than antibiotics, reinforcing its biosafety profile. These findings demonstrate that *L. lactis* can serve as an effective mucosal delivery platform for conserved spike protein antigens, offering a non-invasive and broadly protective vaccination strategy against SARS-CoV-2 and potentially other coronaviruses [147]. Further study of the first vaccine could contribute to the pandemic during 2020.

### 5.2. Bacterial Vaccines

*Clostridium difficile* infection is a leading cause of antibiotic-associated diarrhea. Vaccines targeting the toxins TcdA and TcdB have been evaluated using *L. lactis* expressing these antigens. Guo et al. compared oral *L. lactis* vaccines expressing single or combined toxins with purified recombinant proteins, finding significantly improved survival and reduced pathology in vaccinated animals. All vaccine groups elicited strong IgG and IgA responses with toxin-neutralizing activity, supporting *L. lactis* as a cost-effective oral vaccine platform against *C. difficile* infection [148].

*Helicobacter pylori* is one of the leading causes of gastritis, ulcers, and gastric cancer [149,150]. Initial *L. lactis*-based vaccines expressed the urease B subunit (UreB), eliciting systemic antibody responses with multi-dose oral regimens [150]. Later studies focused on Th1/Th17 immune induction, using antigens like the neutrophil-activating protein (NapA) and the CagL protein, demonstrating antigen-specific antibodies and cytokine responses linked to protective immunity [32,151].

*Streptococcus pneumoniae* is one of the leading pathogens responsible for respiratory diseases worldwide [152]. Although polysaccharide-based vaccines are available, they have the limitation of not providing immunity against all serotypes of this bacterium [153,154,155]. On the other hand, conjugate vaccines offer broader protection but are costly, which can limit their accessibility in low-income countries [156,157]. In this context, the use of *L. lactis* provides a promising alternative to overcome this economic barrier. A study developed a vaccine model using *L. lactis* engineered to express pneumococcal protective protein A (PppA) on its surface, a protein conserved in serotypes 3, 5, 9, 14, 19, and 23. Different respiratory mucosal immunization protocols were evaluated, including nasal administration of live or inactivated *L. lactis* expressing PppA, with or without co-administration of a probiotic (*L. casei*). The mice that received the *L. casei* both orally and nasally showed the highest levels of anti-PppA IgA and IgG antibodies in bronchoalveolar lavage (BAL) fluid and IgG in serum, respectively, which contributed to protection against infection. However, only the groups that received the live or inactivated vaccine together with oral probiotic administration were able to prevent lung colonization by *S. pneumoniae* serotypes 3 and 14 in a respiratory infection model. This protection was associated with a preferential stimulation of local and systemic T helper type 1 (Th1) responses, accompanied by moderate Th2 and Th17 activity, as indicated by cytokine profiles in BAL and the IgG1/IgG2a ratio both locally and systemically. Overall, nasal immunization with the inactivated recombinant strain combined with oral probiotic administration effectively stimulated specific cellular and humoral immune responses, protecting against challenge with the two *S. pneumoniae* serotypes [158].

Enterotoxigenic *Escherichia coli* can cause diarrhea in children, and suitable vaccines are therefore desired [159]. A study evaluated the immune response after the oral and subcutaneous administration of *L. lactis* capable of expressing a heat-labile toxin (LTB), a virulence factor, and compared it with the recombinant protein in a rabbit animal model. They demonstrated an increase in IgA levels in the intestine. An in vitro neutralization assay showed that the toxin’s effect could be neutralized with 500 µg/mL of IgG isolated from the oral vaccine group. Furthermore, the dose of enterotoxigenic *E. coli* causing fluid accumulation in the ileal loop test showed a tenfold increase in rabbits immunized with either recombinant *L. lactis* or LTB protein compared to other groups [160].

*Brucella abortus* is a facultative intracellular, Gram-negative bacterial pathogen that primarily infects humans and animals through the digestive tract [161]. *B. abortus* causes abortion in pregnant cattle and undulant fever in humans [162,163]. *B. abortus* ribosomal antigen L7/L12, a well-characterized immunogenic protein, was expressed under the nisin-inducible PnisA promoter in *L. lactis* NZ9000. Through a series of genetic constructs, the antigen was successfully targeted to three cellular locations (the cytoplasmic, secreted, and cell wall-anchored) using combinations of secretion signals, fusion partners, and anchoring domains. The fusion of L7/L12 with the Usp45 signal peptide enabled secretion with a sixfold higher yield than cytoplasmic production. At the same time, the addition of the LEISSTCDA synthetic propeptide or staphylococcal nuclease (Nuc) further increased yield and secretion efficiency up to 50%. Additionally, anchoring L7/L12 to the M6 protein cell-wall domain from *Streptococcus pyogenes* resulted in stable surface localization, making it particularly suitable for mucosal delivery. This work demonstrated that *L. lactis* can be engineered to produce and export a protective *Brucella* antigen in different cellular compartments, establishing the conceptual foundation for safe, food-grade, non-pathogenic mucosal vaccines against brucellosis and other intracellular bacterial infections [164]. The same authors observed significant levels of anti-L7/L12-specific IgA in feces, revealing an induced local humoral immune response. However, serum analysis did not reveal any anti-L7/L12 antibodies, suggesting the absence of a systemic response [129].

*Streptococcus agalactiae*, also known as Group B *Streptococcus* (GBS), is a leading cause of neonatal sepsis and meningitis, with no licensed vaccine available. The Surface Immunogenic Protein (SIP), conserved across serotypes, is a promising target [165,166,167]. Oral immunization of mice with this recombinant strain elicited strong systemic and mucosal anti-SIP IgG and IgA responses, enhanced CD4^+^ and CD8^+^ T-cell activation, and reduced the frequencies of Treg cells (CD4^+^CD25^+^FoxP3^+^), consistent with a Th1/Th17-oriented immune profile. Notably, vaccinated animals showed a significant reduction in vaginal GBS colonization, and passive transfer of serum or T cells conferred protection to naive mice, confirming the involvement of both humoral and cellular mechanisms. Collectively, these findings demonstrate that *L. lactis* can serve as a safe and effective mucosal delivery platform for the SIP antigen, providing a foundation for non-invasive and broadly protective vaccine strategies against GBS [168]. The same methods were used in a tilapia model with the SIP and a truncated SIP (tSIP). Fish immunized with the tSIP vaccine also showed the highest level of protection compared to other test groups, and the mortality rate was significantly reduced compared to both control groups. The relative percentage of survival (RPS) against *S. agalactiae* was 50% and 89%, respectively, for both SIP and tSIP-vaccinated groups at 14 days post-challenge. Significant up-regulation of IgM, IL-1β, IL-10, TNF-α, and IFN-γ was observed at day 34 between the vaccinated and control groups. These results indicated that the recombinant *lactococcal* tSIP vaccine can elicit both cell-mediated and humoral responses and is recommended as a potential oral vaccine against *S. agalactiae* infection [169].

In summary, *L. lactis* has been widely used for the development of vaccine prototypes against various viral and bacterial pathogens of public health importance. All key aspects related to antigens, administration routes, and main findings are described in Table 2.

## 6. Probiotic, Vaccine, or Drug? Regulations, New Technologies, and Solutions

To date, clinical studies using *L. lactis* as a recombinant bacterial vector have been limited to trials targeting human papillomavirus (HPV), specifically employing the E7 [136] and E6 [171] oncoproteins. Both studies demonstrated the safety profile of the vaccine candidates and their capacity to induce a humoral immune response against the target antigens. These clinical outcomes (IRCT20190504043464N1 and IRCT20190504043464N1, respectively) were supported by prior preclinical evidence from murine models [135,170]. However, both clinical trials utilized *L. lactis* strains transformed with the plasmid pNZ8123, which harbors a chloramphenicol resistance gene as a selection marker [43]. This raises significant biosafety concerns, as plasmids carrying antibiotic resistance genes pose the risk of horizontal gene transfer to commensal or pathogenic bacteria in the host microbiota or environment. Studies have documented gene transfer events from *Lactobacillus* spp. to *E. coli* [172] and other in vivo models [173], underscoring the importance of avoiding antibiotic resistance markers in genetically modified probiotics [174].

The World Health Organization (WHO), in its “Guidelines on Nonclinical Evaluation of Vaccines” (2005), defines vaccines as a heterogeneous class of medicinal products containing immunogenic substances capable of inducing specific, active, and protective host immunity against infectious diseases. When live attenuated vaccines are based on genetically modified organisms (GMOs), the guidelines recommend environmental risk assessments during preclinical development. This includes investigating the potential shedding of vaccine strains after administration and evaluating the risk of genetic exchange with non-vaccine strains, a crucial aspect considering evidence of plasmid transfer among related bacteria [175].

In addition, to the general safety concerns associated with the use of antibiotic resistance markers in GMOs, international regulations impose strict guidelines for the clinical and environmental evaluation of recombinant bacteria in the European Union. *L. lactis* expressing heterologous genes is considered a GMO and is regulated under Regulation (EC) No. 1829/2003, requiring a comprehensive environmental risk assessment, traceability, and labeling before approval [176]. The U.S. regulatory framework is divided among the FDA, USDA, and EPA, depending on the nature of the product and its potential environmental or therapeutic impact.

Furthermore, the regulatory classification of *L. lactis*-based products as probiotics, vaccines, or drugs remains ambiguous. Venugopalan et al. underscore that probiotics may be regulated either as dietary supplements or as drugs, depending on their intended use [177]. The FDA defines a drug as “an article intended for use in the diagnosis, cure, mitigation, treatment, or prevention of disease.” [13]. If a product is marketed as a dietary supplement, it falls under food regulations, which are generally less stringent than those for pharmaceuticals or vaccines. Conversely, suppose the probiotic or recombinant microorganism is intended as a drug or vaccine. In that case, it must comply with rigorous regulatory pathways specific to each country, ensuring evidence of safety, efficacy, and quality through preclinical and clinical studies.

A critical consideration for the development or modification of lactic acid bacteria expression systems is compliance with food-grade safety standards. In the United States, the Food and Drug Administration (FDA) regulates microorganisms used in food under the Generally Recognized as Safe (GRAS) framework, whereby a microorganism or its use must be supported by a substantial history of safe use or adequate scientific evidence to be considered safe under the intended conditions of use, either through expert consensus or formal GRAS notification procedures [178]. In the European Union, the European Food Safety Authority (EFSA) employs the Qualified Presumption of Safety (QPS) approach as a pre-market safety assessment for microorganisms intentionally added to food or feed; this process evaluates taxonomic identity, body of knowledge, and absence of safety concerns, and QPS status can streamline regulatory review if criteria are met [179]. For genetically modified LAB intended for food-grade applications, additional requirements typically include the absence of transferable antibiotic resistance genes, demonstrable lack of pathogenic traits, and robust strain characterization. Given the evolving landscape of live biotherapeutic products (LBPs) such as genetically engineered *L. lactis*, there is an urgent need for harmonized regulatory frameworks that address their unique characteristics. This includes establishing standardized guidelines for genetic modification techniques (e.g., marker-free systems), environmental risk assessment, manufacturing quality control, and post-market surveillance. Such regulatory clarity will facilitate the translation of *L. lactis*-based vaccines and therapeutics from research to clinical use while safeguarding public health and environmental safety.

In recent years, emerging technologies have significantly advanced the development of genetically modified *L. lactis*-based vaccines. The CRISPR-Cas system enables precise genomic modifications, allowing gene insertion without the use of antibiotic resistance markers or mobile genetic elements, thereby facilitating compliance with biosafety requirements established by national and international regulatory agencies such as the WHO, FDA, and EFSA. Moreover, several studies have already reported the successful application of this technology in *L. lactis* [180,181,182] In addition, bacterial microencapsulation has been employed as a complementary strategy not only to enhance immune responses and improve cell viability throughout the gastrointestinal tract [183,184] but also as a biocontainment measure to prevent the environmental dissemination of the microorganism [185,186].

## 7. Learnings and New Perspectives

Over the past decade, numerous reports have highlighted the potential of oral vaccines based on *L. lactis*, demonstrating both protective efficacy and the induction of humoral and cellular immune responses. However, much of this evidence remains confined to plasmid-based platforms and preclinical stages, limiting scalability and clinical translation despite promising results. We consider it a priority to promote the development of genetically modified organisms (GMOs) with stable genomic integration as a necessary step to meet regulatory requirements and facilitate the transition to clinical studies. In parallel, we urge regulatory authorities to establish harmonized, step-by-step guidelines for evaluating genetically modified probiotics, encompassing biosafety, environmental risk assessment, traceability, mitigation plans, and requirements for release and monitoring. Given the potential of these platforms not only for vaccine development but also for therapeutic applications in cancer, allergies, and other conditions, the availability of clear and up-to-date regulatory frameworks is essential to accelerate their responsible and safe advancement.

## 8. Conclusions

Genetically engineered *L. lactis* has evolved from a safe food-grade bacterium to a versatile live biotherapeutic platform capable of delivering antigens, cytokines, and therapeutic molecules at mucosal surfaces. Its well-characterized genetics, absence of endotoxins, and the availability of finely tunable expression systems such as NICE, XIES, zinc-responsive, and stress-inducible promoters have positioned it as one of the most promising candidates for next-generation mucosal vaccines. Preclinical studies have demonstrated its ability to elicit both mucosal and systemic immune responses. However, the clinical translation of *L. lactis*-based vaccines remains limited by regulatory uncertainty, concerns regarding biosafety and antibiotic resistance markers, as well as the need for standardized manufacturing and evaluation guidelines. Advances in marker-free genome editing, such as CRISPR-Cas, and biocontainment technologies, including bacterial microencapsulation, offer practical solutions to these challenges, paving the way for the safe and effective deployment of *L. lactis* as a live vaccine vector. Ultimately, integrating these innovations within harmonized international regulatory frameworks will be essential to fully realizing the potential of *L. lactis* as a new strategy for mucosal immunization and the design of live biotherapeutics.

## Figures and Tables

**Table 2:** Mucosal Vaccines based on *L. lactis* expressing heterologous antigens.

Pathogen	Antigen	Route of Administration	Response	References
HPV	E7 Oncoprotein	Oral	Induced E7-specific IgG antibodies and activation of CD4^+^ T cells; demonstrated therapeutic potential confirmed in early-phase clinical trials	[135,136,170]
HIV	Gag–T3 pilus fusion; V2–V4 Env loop	Oral	Increased Gag-specific IgG and IgA in serum, feces, and vaginal secretions; activation of dendritic cells in Peyer’s patches; limited CD8^+^ T-cell response	[137,138,139]
Influenza A	Hemagglutinin (HA) and HA-stalk fusion	Oral	Induced strong serum IgG and mucosal IgA responses; conferred complete protection in mice and cross-protection among influenza subtypes	[141,142]
HBV	PreS region or central HBsAg fragments ± IFN-γ	Oral	Elicited serum IgG and intestinal IgA; co-expression of IFN-γ enhanced the humoral response	[143,144]
SARS	Nucleocapsid (N) protein; RBD; HCR (S2 subunit)	Oral/Intranasal	Induced anti-SARS-CoV-2 IgG and IgA responses; activated CD4^+^ and CD8^+^ T cells; stronger NALT response via intranasal route; developed under food-grade conditions	[145,146,147]
*C. difficile*	Toxin fragments TcdA, TcdB	Oral	Elicited high IgG and IgA levels with toxin-neutralizing activity; improved animal survival and reduced intestinal pathology	[148]
*H. pylori*	UreB; NapA; CagL	Oral	Induced antigen-specific IgG and mucosal IgA; promoted a Th1/Th17 cytokine profile associated with protection	[32,150,151]
*S. pneumoniae*	Pneumococcal protective protein A (PppA)	Oral/intranasal	Increased IgA and IgG in bronchoalveolar lavage and serum; conferred protection against serotypes 3 and 14; promoted Th1-dominant response with moderate Th2/Th17 activity	[158]
*E. coli*	Heat-labile toxin subunit B (LTB)	Oral/subcutaneous	Induced intestinal IgA and serum IgG; demonstrated in vitro toxin neutralization; provided protection in the rabbit ileal loop assay	[160]
*Brucella abortus*	Ribosomal protein L7/L12	Oral	Induced fecal IgA indicating local response; no systemic IgG detected; secretion improved with Usp45 and LEISS pro-peptide; anchored form enhanced mucosal delivery	[164]
*C. difficile*	Toxin fragments TcdA, TcdB	Oral	Elicited high IgG and IgA levels with toxin-neutralizing activity; improved animal survival and reduced intestinal pathology	[148]
*S. agalactiae*	SIP	Oral	Strong systemic and mucosal anti-SIP IgG and IgA responses; enhanced CD4^+^ and CD8^+^ T-cell activation	[168]

## Data Availability

The original contributions presented in this study are included in the article. Further inquiries can be directed to the corresponding author.

## Abbreviations

The following abbreviations are used in this manuscript:

APCs	Antigen-Presenting Cells
BALT	Bronchus-Associated Lymphoid Tissue
BAL	Bronchoalveolar Lavage
CD	Cluster of Differentiation (CD4, CD8, etc.)
CFU	Colony-Forming Units
CTL	Cytotoxic T Lymphocyte
CTLA-4	Cytotoxic T-Lymphocyte Antigen 4
DCs	Dendritic Cells
EPA	Environmental Protection Agency
FDA	Food and Drug Administration
GALT	Gut-Associated Lymphoid Tissue
GFP	Green Fluorescent Protein
GMOs	Genetically Modified Organisms
GOALTs	Genital Organ-Associated Lymphoid Tissues
GRAS	Generally Recognized As Safe
GST	Glutathione-S-Transferase
HA	Hemagglutinin
HBV	Hepatitis B Virus
HBsAg	Hepatitis B Surface Antigen
HCR	Highly Conserved Region
HIV	Human Immunodeficiency Virus
HPV	Human Papillomavirus
IL	Interleukin
IRCT	Iranian Registry of Clinical Trials
LBPs	Live Biotherapeutic Products
LPS	Lipopolysaccharide
LTB	Heat-Labile Toxin Subunit B
MALT	Mucosa-Associated Lymphoid Tissue
MHC	Major Histocompatibility Complex
NALT	Nasal-Associated Lymphoid Tissue
NICE	Nisin-Controlled Gene Expression system
OD	Optical Density
PnisA	Nisin-Inducible Promoter A
PppA	Pneumococcal Protective Protein A
PxylT	Xylose-Inducible Promoter
BD	Receptor-Binding Domain
RPS	Relative Percent Survival
SALT	Skin-Associated Lymphoid Tissue
SARS	Severe Acute Respiratory Syndrome
SICE	Stress-Inducible Controlled Expression system
SIgA	Secretory Immunoglobulin A
SIP	Surface Immunogenic Protein
TcdA	Clostridioides difficile Toxin A
TcdB	Clostridioides difficile Toxin B
Tregs	Regulatory T Cells
Usp45	Universal Stress Protein 45 (signal peptide)
VLPs	Virus-Like Particles
WHO	World Health Organization
XIES	Xylose-Inducible Expression System
ZICE	Zinc-Controlled Expression system
Zirex	Zinc-Inducible Regulatory Expression system
